# Reconfigurable and optically transparent microwave absorbers based on deep eutectic solvent-gated graphene

**DOI:** 10.1038/s41598-019-41806-w

**Published:** 2019-04-02

**Authors:** Marco Grande, Giuseppe Valerio Bianco, Filippo Maria Perna, Vito Capriati, Pio Capezzuto, Michael Scalora, Giovanni Bruno, Antonella D’Orazio

**Affiliations:** 1 Dipartimento di Ingegneria Elettrica e dell’Informazione, Politecnico di Bari, Via Re David 200, 70125 Bari, Italy; 2 Istituto di Nanotecnologia – CNR-NANOTEC, Via Orabona, 4, 70125 Bari, Italy; 3 Dipartimento di Farmacia-Scienze del Farmaco, Università di Bari “Aldo Moro”, Consorzio C.I.N.M.P.I.S., Via E. Orabona 4, 70125 Bari, Italy; 4 Charles M. Bowden Research Center, RDECOM, Redstone Arsenal, Alabama, 35898-5000 USA

## Abstract

Electrolytically tunable graphene “building blocks” for reconfigurable and optically transparent microwave surfaces and absorbers have been designed and fabricated by exploiting Deep Eutectic Solvents (DESs). DESs have been first explored as electrolytic and environmentally friendly media for tuning sheet resistance and Fermi level of graphene together with its microwave response (reflection, transmission and absorption). We consider the tunability of the reconfigurable surfaces in terms of transmittance, absorption and reflectance, respectively, over the X and Ku bands when the gate voltage is varied in the −1.4/+1.4 V range. The numerical simulations and experimental measurements also show the ability of the absorber, in the Salisbury screen configuration, to achieve near perfect absorption with a modulation of about 20%. These results could find applications in several technological fields, ranging from electromagnetic pollution to integrated multi-physical regulation systems, thereby helping the advance of the performance of microwave cloaking systems, stealth windows, frequency selective surfaces, modulators and polarizers.

## Introduction

The quest for optically transparent microwave devices has increased over the last years. This growing research interest is driven by the opportunity to combine photonic and microwave technologies, thus creating new applications^1,2^. In this framework, one important aspect is related to the ability to manipulate and control reflection, transmission and absorption of microwave radiation in devices that are also optically transparent. In the literature, there are recent examples where attempts are made to achieve microwave absorption in optically transparent structures by means of several configurations based on metamaterials^3–9^, metasurfaces^10^, metallic meshes^11,12^, and Salisbury screens^13^. Recently, the possibility to integrate an optically transparent absorber with an acoustic absorber has also been reported^14^, leading to a multi-physical regulation system. These devices offer new functionalities, although they share a common limitation: they are “static” and their properties mainly relate to their geometry.

Up to now, few reconfigurable examples of microwave absorbers based on electrolyte-gated graphene have been proposed^15–17^. Electrolytic gating allows the controlled switching from pristine (high Rs) to doped graphene (low Rs). This opens the possibility (for an optimized configuration) to switch from a microwave transparent window to an absorbing or reflecting one. However, the reported examples of microwave absorbers typically have configurations based on metallic and opaque surfaces. Thus, they are not transparent in the visible range. Moreover, electrolytic gating of graphene typically makes use of ionic liquids (especially of imidazolium-derived ionic liquids)^18,19^ which have several drawbacks in terms of cost and toxicity^20^. This strongly limits their potential applications in commercial devices.

In this paper, we take a step forward on the path towards innovative and reconfigurable “optically transparent” graphene-based devices, overcoming both limitations. We propose an optically transparent, flexible and reconfigurable microwave absorber using two main strategies/approaches: (i) an electrolyte-gated graphene capacitor exploiting a Deep Eutectic Solvents (DES) as electrolytic medium and (ii) a “quasi-metal” graphene microwave mirror. In particular, we first propose DESs as electrolytic media for tuning sheet resistance and Fermi level of graphene in order to fully control its microwave response (reflection, transmission and absorption). DESs, which share several physical-chemical properties with traditional ionic liquids (e.g., low vapour pressure, thermal stability, non-flammability, easy of recycling), are biodegradable fluids usually made by mixing and gently heating two or three safe and inexpensive components. DES components, often solid compounds at room temperature, are able to interact with each other mainly through hydrogen-bonding interactions, thereby forming eutectic mixtures whose eutectic point temperature is far below those of the individual components. Common mixtures derive from a quaternary ammonium salt (e.g. choline chloride, ChCl) and a neutral hydrogen-bond donor such as glycerol (Gly), urea and natural carboxylic acids. Because of their minimal ecological footprint, in the last few years DESs have been progressively replacing common and toxic volatile organic compounds in several fundamental and applied processes^21,22^. They have recently gained increasing attention, in particular in regard to metal-catalysis and organometallics^23^, energy technology^24^, electrochemistry^25^, and nanomaterials growth^26^, with surprising and unexpected results. In what follows, we demonstrate that DESs represent a valid, low-cost alternative to ionic liquids for realizing a graphene capacitor with a suitable electrochemical window. Specifically, we investigated the hydrophilic ChCl/Gly (1:2 mol/mol) eutectic mixture (melting point −35 °C) as the electrolytic medium. ChCl, also known as vitamin B_4_, is one of the most widely used ammonium salts extracted from biomass or synthesized from fossil reserves. Thus, it is a very cheap (ca. 2€ kg^−1^) and biodegradable material, and is produced on the scale of a million metric tonnes per year.

On the other hand, the realization of “quasi-metal” graphene microwave mirror takes advantage of the chemical protocol based on SOCl_2_ that allows the realization of graphene sheets with very low sheet resistance (down to less than 20 ohm/sq)^27^ that can be implemented as efficient microwave mirror (supplying the need of metallic components)^13^.

We thus show that microwave transmittance, reflectance and absorption of the graphene capacitor using the above ChCl-based eutectic mixture can be varied by about 24%, 10% and 15% over the X and Ku bands when an electrochemical window in the −1.4/+1.4 V range is considered. Finally, we numerically and experimentally prove that the DES-gated graphene capacitor can be integrated in a Salisbury screen configuration leading to near perfect absorption with a modulation of about 20%. These results may open the floodgates to unprecedented fully transparent devices for several applications ranging from multi-physics regulation, cloaking, to stealth and other systems.

## Results and Discussion

**Figure 1:**
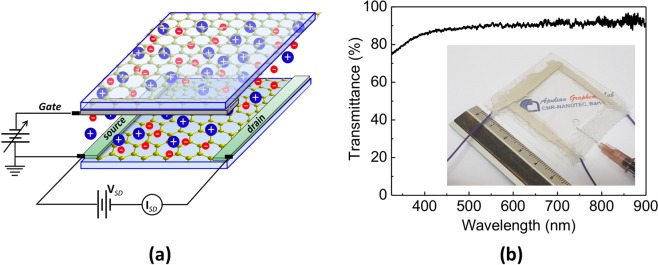
(**a**) Sketch of graphene electrolytic capacitor. By applying gate voltage, carriers of opposite signs are accumulated on graphene electrodes whose Fermi levels are shifted in valence and conduction band. (**b**) Optical transmittance of the graphene electrolytic capacitor. Inset: picture of the fabricated capacitor.

Figure 1(a) shows the sketch of the proposed graphene-based electrolytic capacitor consisting of two CVD graphene electrodes on PET substrates and electrolyte medium between them. When a voltage is applied between the graphene electrodes, the electrolytic medium polarizes, and ionic double layers are formed at the graphene–electrolyte interfaces. Thus, carriers of opposite signs are accumulated on graphene electrodes whose Fermi levels are shifted in valence (*p*-doped) and conduction band (*n*-doped), respectively.

The modulation range of graphene electrodes is determined by both graphene structural quality and electrolyte type. The specific electrochemical window of the electrolyte (i.e., the voltage range between which the substance is neither oxidized nor reduced) defines the maximum charge density that can be injected in graphene. On the other side, the quality of graphene electrodes limits the minimum Rs value achievable within the electrochemical windows. We realized an electrolytic capacitor based on double-layer (2L) graphene electrodes. Figure 1(b) shows the transparency of the fabricated device, which is higher than 90% (λ > 550 nm).

**Figure 2:**
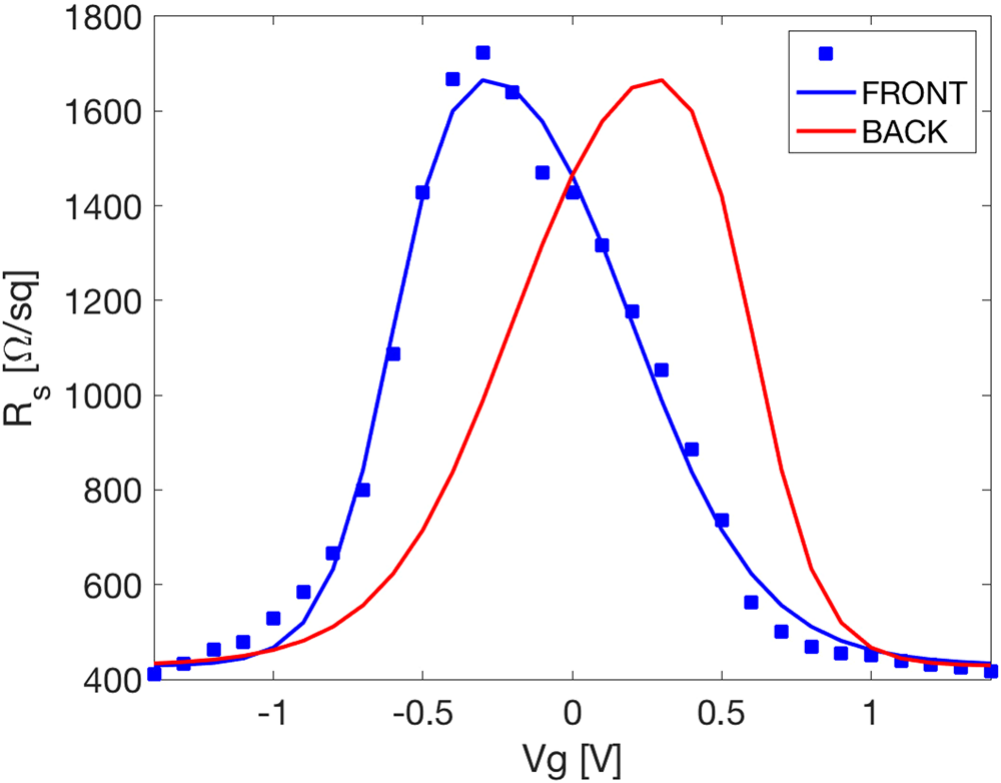
Experimental Rs values (blue points) of 2L graphene front electrode as a function of the applied gate voltage. The blue curve represents the fitting of experimental Rs data by means of an asymmetric double sigmoidal function. Red line represents the expected mirrored trend of Rs for the 2L back electrode.

The inset in Fig. 1(b) shows the fabricated graphene capacitor during filling with ChCl-based DES. Figure 2 shows the graphene sheet resistance of the graphene-based electrodes of the capacitor. In particular, the blue points in Fig. 2 indicate the Rs values of front graphene electrode of our electrolytic capacitors as a function of applied gate voltage Vg in the field effect configuration. Conversely, blue and red lines in Fig. 2 represent, respectively, the fitting of experimental Rs data (by means of an asymmetric double sigmoidal function) and the expected mirror Rs trend for graphene electrodes since charges of opposite signs accumulate on front and back graphene electrodes under gate voltage.

The voltage at which the resistivity reaches the maximum value is defined as Dirac voltage, V_D_. A V_D_ located at positive or negative voltages is indicative of an intrinsic *p-* or *n-*doping state. This intrinsic doping can result from graphene chemical defects and charge transfer from the substrate, the electrolytic medium and charged impurities adsorbed on graphene. The 2L electrode reaches the maximum resistivity value at −0.3 V. When a V_g_ equal to V_D_ is applied, the Fermi Level is located at the Dirac point, and the material presents the minimum carrier concentration. Then, for V_g_ > V_D_ or V_g_ < V_D_, the material becomes electrically *n-* or *p-*doped, respectively, and its Rs decreases. In the exploited electrochemical window (from −1.4 to 1.4 V), the 2L electrode, the Rs modulation range is shifted in the 1700-416 ohm/sq range with a modulation ratio around 4. It is worth noting that the electrochemical window is fixed in the −1.4 to 1.4 V range in order to avoid electrolyte oxidation or reduction at electrode surfaces.

**Figure 3:**
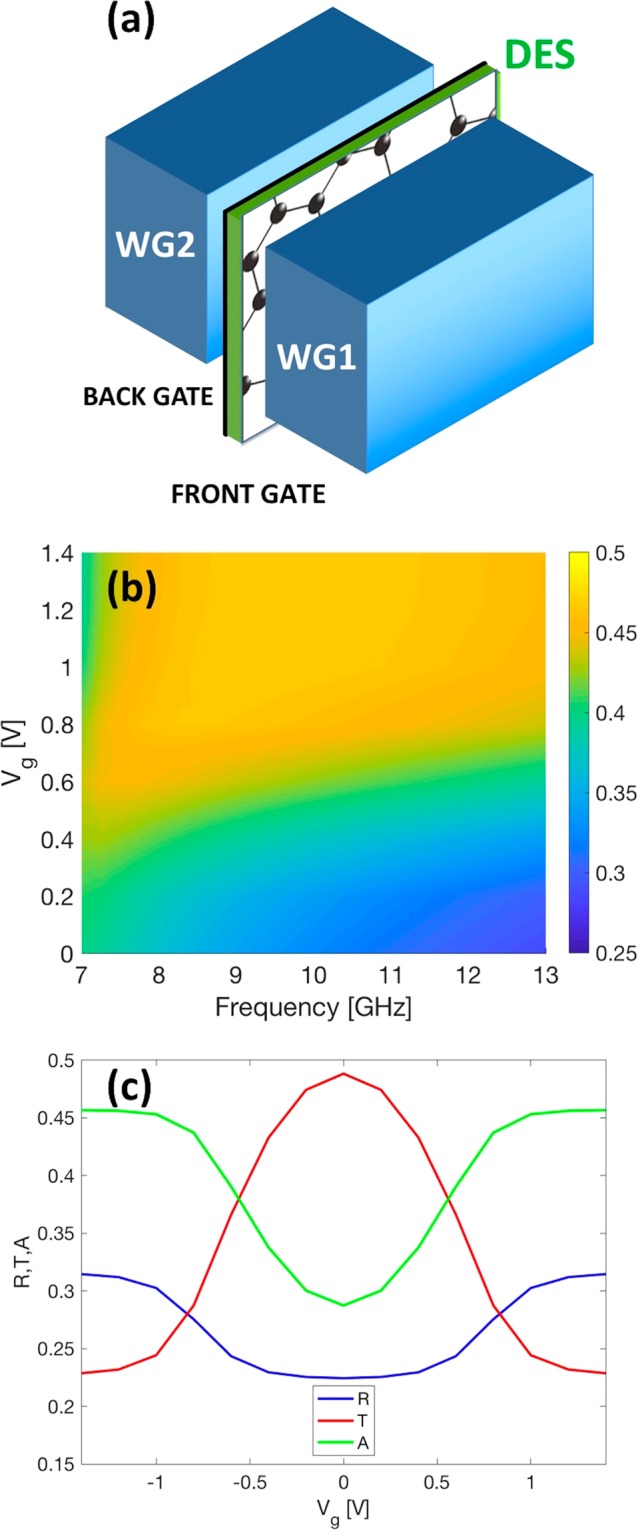
(**a**) Sketch of the numerical model consisting of the graphene-based capacitor sandwiched between two WR90 waveguides (WG1 and WG2). (**b**) Numerical absorption map in the frequency range of interest. (**c**) Numerical reflectance, transmittance and absorbance when the gate voltage is varied in the DES electrochemical window at 13 GHz.

To assess the performance of graphene-base (or G-based) capacitor in the microwave range, we developed a 3D numerical model by means of Comsol-Multiphysics. Figure 3(a) shows the sketch of the G-based capacitor sandwiched between two WR-90 waveguides (WR, Waveguide Rectangular). The front and the back electrodes were modelled by two surface currents (J =  σ * E) with different sheet resistance (details in “Simulations” section)^28^.

We consider mono-modal propagation in the metallic rectangular waveguide and limit our analysis to the frequency range between 7 GHz and 13 GHz (below the cutoff frequency of the TE_20_ mode). We set the permittivity and the thickness of the DES equal to 40 and 100 microns, respectively. The contribution of the PET that supports the graphene in the experiments is neglected since it is almost “transparent” in the frequency range of interest.

Figure 3(b) shows the calculated absorption over the frequency range of interest (7–13 GHz) when the gate voltage is varied in the electrochemical window of interest. The map reveals that the absorption increases with the gate voltage Vg and its maximum moves at higher frequencies. The maximum variation is obtained at higher frequencies, while at lower frequencies, i.e. 7–7.5 GHz, the absorption shows mild modulation. Figure 3(c) shows the calculated reflectance, transmittance and absorbance at 13 GHz when the gate voltage is varied. The plot reveals that by varying the gate voltage in the electrochemical window it is possible to tune transmittance, reflectance and absorption by about 24%, 10% and 15%, respectively. As expected, as the voltage increases transmittance decreases since the graphene moves from a lossy-dielectric towards a quasi-metallic state and the reflectance increases. Overall, absorption increases with the gate voltage as well. Finally, it is worth noting that the plots are symmetric due to the assumption that front and back electrodes are symmetric.

**Figure 4:**
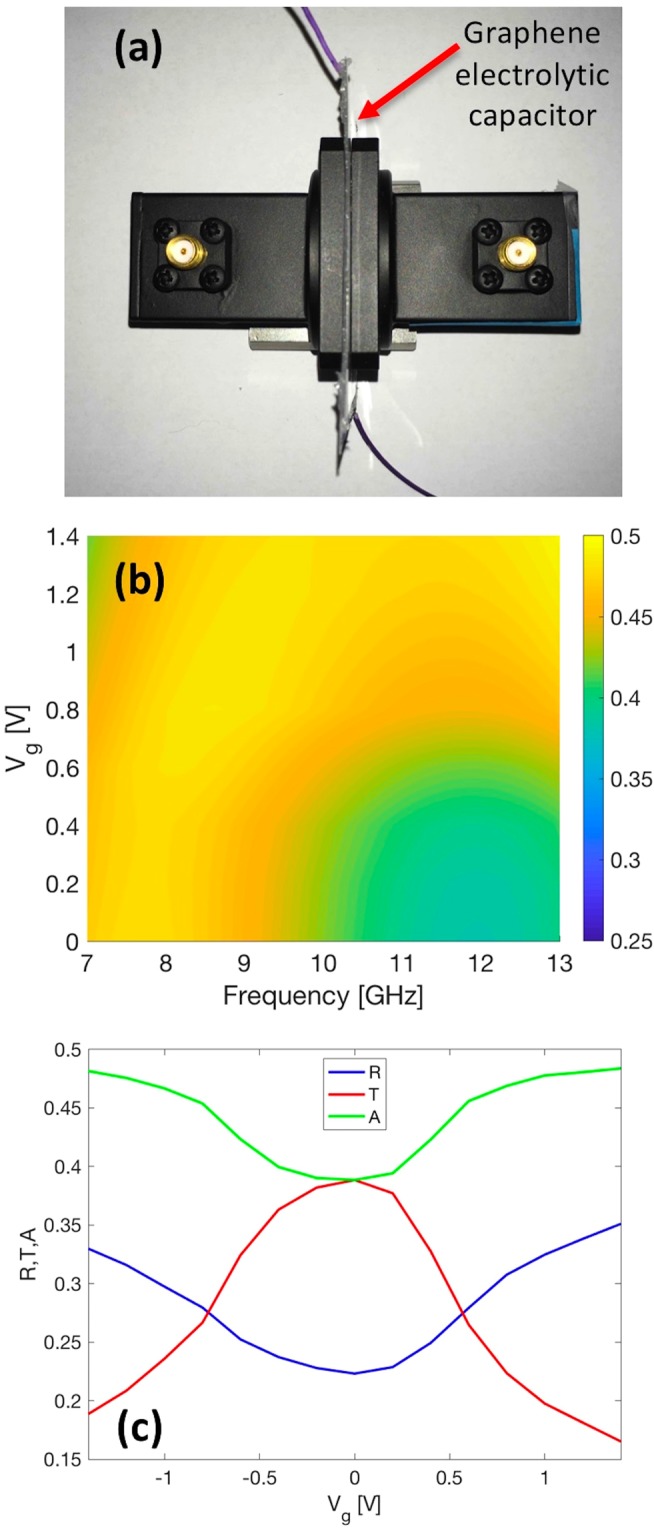
(**a**) Picture of the experiential setup with the two WR-90 transitions that sandwich the graphene electrolytic capacitor. (**b**) Absorption map. (**c**) Experimental reflectance, transmittance and absorbance when the gate voltage is varied in the DES electrochemical window at 13 GHz.

In order to validate the numerical model, we realized an experimental setup (Fig. 4(a)) to measure transmittance, reflectance and absorbance in the frequency range of interest. Two WR90 waveguides were connected to the VNA by means of two SMA/rectangular waveguide transitions. The fabricated G-based capacitor was sandwiched between the two rectangular waveguides (WR1 and WR2). Figure 4(b) details the experimental absorption map while Fig. 4(c) shows reflectance, transmittance and absorbance at 13 GHz. The comparison between Figs 3(b,c) and 4(b,c) shows very good agreement. In particular, the plot in Fig. 4(c) reveals that it is possible to tune transmittance, reflectance and absorption by about 24%, 10% and 15%, respectively. This is fully consistent with the map reported in Fig. 3(c).

Moreover, the experimental transmittance, reflectance and absorption plots at 13 GHz are almost symmetric with respect to Vg = 0 Volts (the error is in the range of 1–2%). These results fully confirm that the difference between the front and bottom electrodes is negligible and, hence, the fitted model of Equations 1 and 2 (simulation) is valid and can be applied. In this case, the curves seem almost symmetric with respect to the central gate voltage (0 V) of the DES electrochemical window, confirming once again our assumption.

**Figure 5:**
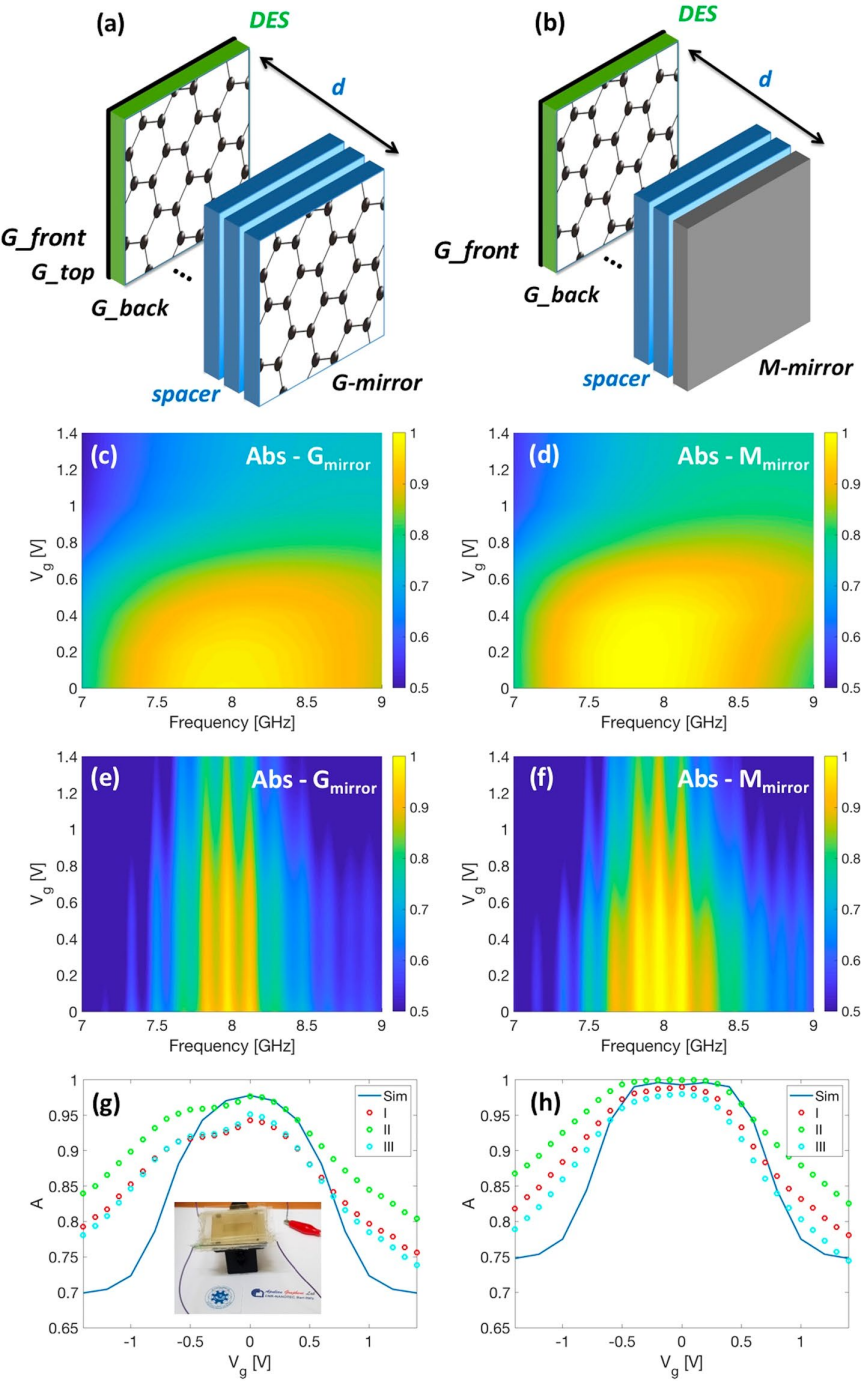
Sketch of the Salisbury screen realized by sandwiching the glass spacer between the electrochemical capacitor and the (**a**) graphene-based and (**b**) metallic mirrors, respectively. In both configurations the spacer thickness d is equal to 3.8 mm in analogy to what has been done in ref.^13^. (**c**,**d**) Theoretical and (**e**,**f**) experimental absorption maps for (**c**–**e**) G-mirror and (**d**–**f**) M-mirror. (**g**,**h**) Absorption versus gate voltage V_g_ for three maxima (I - first at 7.84 GHz, II - second at 7.97 GHz and III -third at 8.11 GHz). The blue line corresponds to the numerical absorption at 7.97 GHz. Inset (**g**) picture of the transparent absorber based on the Salisbury screen configuration.

Finally, the proposed graphene-based capacitor was used to implement an optically transparent Salisbury screen configuration as sketched in Fig. 5(a,b). The Salisbury screen was realized by sandwiching the glass spacer between the electrochemical capacitor and a 6-layer graphene-based and metallic mirror, respectively in analogy to what was done in ref.^13^. Figures 5(c,d) show the numerical absorption maps of the Salisbury screen when the gate voltage is varied in the range of interest for two different mirrors, i.e. graphene and metallic (aluminium) ones. In the first case, we neglect the transmission of the graphene film since the transmission for this film (Rs equal to 20 ohm/sq) is very low^13^. Therefore, we determined the absorption A = 1 − R (R = |S_11_|^2^) in both cases. Both numerical maps show that the absorption of the Salisbury screen reaches near perfect absorption when Vg = 0 V at about 8 GHz. As Vg increases, the absorption drops down by about 25% (to the detriment of the reflection). Experimental results reported in Fig. 5(e,f) are in good agreement with the numerical simulations, showing asymmetrical behaviour where the absorption band is extended to higher frequencies (>8 GHz). The ripples are mainly due to the high frequency resolution used in the measurement (2 MHz). The numerical and the experimental results are depicted and compared each other in Fig. 5(g,h). For experimental data, we considered the absorption corresponding to the three main maxima indicated with I (left), II (central) and III (right). All these plots confirm good agreement between experimental data and the numerical model.

Maximum absorption of about 97.80% and 99.98% is achieved for the graphene-based and metallic mirrors, respectively. When the absorption higher than 90% is considered, Fig. 5 allows to retrieve theoretical and experimental bandwidths that are equal to about 1.4 GHz for both graphene-based and metallic mirrors. At the same time, absorption modulation of about 20%, in both numerical and experimental measurements, is reported. The optical transparency of the complete absorber is above 75% at 550 nm.

## Conclusions

In this paper, we demonstrate the possibility to realize reconfigurable and optically transparent microwave absorbers. This goal was achieved by exploiting highly-doped graphene mirrors operating in the quasi-metallic region and DES-gated graphene capacitors. The combination of these two building blocks allowed us to realize reconfigurable (i) surfaces and (ii) absorbers. Numerical and experimental findings show the ability of these two devices to nearly fully control microwave transmittance, reflectance and absorption of graphene over the X and Ku bands. It is worth highlighting that the independent behaviour of the graphene in the microwave region^28^ allows us to extend these concepts to different microwave frequencies and bands.

Therefore, these results are a mere first step towards innovative and reconfigurable, optically-transparent, graphene-based devices. The flexibility of the proposed surface on flexible substrates could be useful for several applications, such as cloaking and electromagnetic anti-pollution systems, modulators and polarizers. We believe these findings pave the way to conceive a plethora of innovative devices that can simultaneously work in the photonic and microwave domains.

## Methods

### Simulations

To assess the performance of the G-based capacitors at microwave, we realized a 3D numerical model by means of Comsol-Multiphysics. Considering that the thickness of a single graphene layer is 3.4 Å, a few-layer (1–6 layers) graphene sample is around 1 nm-thick that corresponds to ∼λ/10^6^ in the L-S-C-X microwave bands^28,29^. This is effectively enough to consider both single and few-layer graphene as an interface with a current sheet J (modelled as J = Ex, y/Rs). In turn, the microwave absorption only depends on the Rs of graphene sample rather than their thickness. Therefore, the front and the back electrodes were modelled by two surface currents (J =  σ * E) with different sheet resistance^13^. In particular, Rs values of both front and back electrodes were fitted by means of an asymmetric double sigmoidal function as follows (solid lines in Fig. 2):

1 $$R{s}_{top}={y}_{0}+{\rm{A}}(\frac{1}{1+{e}^{-\frac{{V}_{g}-{x}_{c}+{w}_{1}/2}{{w}_{2}}}})(1-\frac{1}{1+{e}^{-\frac{{V}_{g}-{x}_{c}-{w}_{1}/2}{{w}_{3}}}})$$

2 $$R{s}_{back}={y}_{0}+{\rm{A}}(\frac{1}{1+{e}^{-\frac{-{V}_{g}-{x}_{c}+{w}_{1}/2}{{w}_{2}}}})(1-\frac{1}{1+{e}^{-\frac{-{V}_{g}-{x}_{c}-{w}_{1}/2}{{w}_{3}}}})$$

where y_0_ = 428.32763, A = 1445.88832, x_c_ = −0.19967, w_1_ = 0.80239 w_2_ = 0.1112, and w_3_ = 0.21377.

As is evident from Eqs (1) and (2), it is worth stressing that we are supposing that the front and back electrodes show symmetric behaviour with respect to V_g_ = 0 V. This assumption has been verified in the experimental section (“Results and discussion”).

### Material Synthesis And Characterization

Graphene was grown by chemical vapor deposition methodology on 25 μm thick copper foils at 1000 °C using CH_4_ and H_2_ as precursors. Copper substrate was etched in solution of ammonium persulfate and thermal tape was used for transferring graphene onto corning glass or PET substrates. SOCl_2_ chemical doping of multilayer-graphene on corning glass was performed in a dry chamber at 105 °C for 60 min (by repeating the same treatment after stacking supplementary graphene layer). Graphene sheet resistance was measured using a four-point contact geometry in the Van der Pauw configuration (5 × 5 mm^2^).

The eutectic mixture of ChCl/glycerol (1:2 mol/mol) was prepared by heating the corresponding individual components to 60 °C under stirring for 10 min until a clear solution was obtained.

### Fabrication And Characterization Of Graphene Capacitors

Graphene front and back electrodes consist of 45 * 45 mm^2^ two-layer graphene foil on 120 µm thick PET foil. Conductive silver paste stripes were used as gate contact (bottom electrode) and source/drain contacts (front electrode). Silicone glue was used to electrically isolate the silver contacts and to seal capacitor. Ag contacts covered with silicone glue (total thickness around 100 µm) act as spacers between the two graphene electrodes. Source and drain contacts with a broad surface area (width above 4 mm) were used in order to minimize contribution of contact resistance during measurements of graphene resistance. Rs of graphene electrode measured by Van der Pauw four-probe system resulted almost equal to Rs value derived by considering source/drain resistance and relative geometric factors. This attested the negligible contribution of contact resistance. Thus, Rs data of graphene electrode as a function of Vg (−1.4 < Vg < 1.4) were derived by source/drain resistance measurements (V_SD_ = 100 mV).

## Data Availability

The datasets generated during and analysed during the current study are available from the corresponding author on reasonable request.
